# Supplementary material for: Solid-phase synthesis of protein-polymers on reversible immobilization supports
Source: Nat Commun. 2018 Feb 27;9:845. doi: 10.1038/s41467-018-03153-8 (PMC5829226; doi:10.1038/s41467-018-03153-8)
Supplement: Supplementary file 1 — Supplementary Information [file 41467_2018_3153_MOESM1_ESM.pdf]

Supplementary Information

Solid-Phase Synthesis of Protein-Polymers

On Reversible Immobilization Supports

Hironobu Murata[1], Sheiliza Carmali[1,4], Stefanie L. Baker[1,2], Krzysztof Matyjaszewski[1,4], Alan J. Russell[1,2,3,4,5*]

[1]Center for Polymer-based Protein Engineering, Carnegie Mellon University, 5000 Forbes Avenue, Pittsburgh, PA 15213, United States

[2]Department of Biomedical Engineering, Scott Hall 4N201, Carnegie Mellon University, 5000 Forbes Avenue, Pittsburgh, PA 15213, United States

[3]Disruptive Health Technology Institute, Carnegie Mellon University, 5000 Forbes Avenue, Pittsburgh, PA 15213, United States

[4]Department of Chemistry, Carnegie Mellon University, 4400 Fifth Avenue, Pittsburgh, PA 15213, United States

[5]Department of Chemical Engineering, Carnegie Mellon University, 5000 Forbes Avenue, Pittsburgh, PA 15213, United States

Supplementary Methods

Instrumentation and Sample Analysis Preparations ^{1}H and ^{13}C NMR were recorded on a spectrometer (300 MHz, 75 MHz, Bruker Avance[TM] 300) in the NMR facility located in Center for Molecular Analysis, Carnegie Mellon University, Pittsburgh, PA, with deuterium oxide (D_2O), DMSO-d_6 and $CDCl_3$. Routine FT-IR spectra were obtained with a Nicolet Magna-IR 560 spectrometer (Thermo), in the Department of Chemical Engineering at Carnegie Mellon University. UV−VIS spectra were obtained and used for enzyme activity determination using a UV−VIS spectrometer (Lambda 2, PerkinElmer) with a temperature-controlled cell holder. Melting points (mp) were measured with a Laboratory Devices Mel-Temp. Number and weight average molecular weights (M_n and M_w) and the polydispersity index (M_w/M_n) were estimated by gel permeation chromatography (GPC) on a Water 2695 Series with a data processor, equipped with three columns (Waters Ultrahydrogel Linier, 500 and 250), using Dulbecco’s Phosphate Buffered Saline with 0.02 wt% sodium azide as an eluent at flow rate 1.0 mL/min, with detection by a refractive index (RI) detector. Pullulan standards (PSS-Polymer Standards Service – USA Inc, Amherst, MA) were used for calibration.Matrix-Assisted Laser Desorption Ionization Time-of-Flight Spectrometry (MALDI-ToF MS) was performed with a Perseptive Biosystems Voyager Elite MALDI-TOF spectrometer in the Center for Molecular Analysis, Carnegie Mellon University. Dynamic Light Scattering (DLS) data were collected on a Malvern Zetasizer nano-ZS, which was located in the Department of Chemistry, Carnegie Mellon University. The concentration of the sample solution was kept at 0.2 - 1.0 mg/mL. The hydrodynamic diameter of samples was measured three times (15 run to each measurement, reported as number distribution) in various buffers.

Preparation of Cu-HMTETA as deoxygenated catalyst solution 100 mM $CuCl_2$ in deionized water (1.2 mL, 120 µmol) was bubbled with N_2 for 25 min and then 100 mM sodium ascorbate in deionized water (120 µL, 12 µmol) was added. HMTETA (39 µL, 144 µmol) was added to the copper suspension bubbled with N_2 for 3 min. The deoxygenated Cu-HMTETA solution was added to the synthesis vessel immediately.

2-Nitro-5-thiocyanobenzoic (NTCB) digestion of protein-initiator conjugates All buffers and reagents were prepared fresh for the NTCB reactions. Native protein or initiator-modified protein complex (10-20 µg) was dissolved in 1 M glycine, 6 M guanidine–HCl pH 10.0 (15 µL) and treated with 1.5 µL of 100 mM of DTT for 5 min at 95 °C. After this time, 22 mM NTCB (20-fold excess of total cysteine content in protein) was added and the digestion was carried out at 37 °C for 18 hours.[50] The reaction was stopped by the addition of 3 µL of TFA and digested samples were purified using ZipTipC_{18} microtips and eluted with 2 µL of matrix solution (20 mg/mL sinapinic acid in 50 % acetonitrile with 0.1 % TFA) directly onto a MALDI-ToF plate for subsequent analysis. The molecular weight of the expected peptide fragments before and after digestion was predicted using PeptideCutter (ExPASy Bioinformatics Portal, Swiss Institute of Bioinformatics). Peptide fragment containing the *N*-terminal group was examined for modification.

Irreversible inactivation of native AChE in low pH (3 – 6) Native AChE (0.4 µM) was dissolved in 20 mM citrate buffer (pH 3 – 6) or 100 mM sodium phosphate buffer (pH 5 and 6) and incubated at room temperature. At given time points, aliquots (10 µL) were removed and activity was measured in 930 µL of 100 mM sodium phosphate buffer (pH 7.4), 50 µL of acetylthiocholine iodide (10 mM in cold 100 mM sodium phosphate buffer (pH 7.4), Sigma Aldrich, St Louis, MO) and 10 µL of 5,5'-dithiobis(2-nitrobenzoic acid) (DTNB, 50 mM in

DMSO, Sigma Aldrich) at 37 °C. The residual activity was calculated as a ratio of initial rates of the reaction at given incubation time over initial activity of native AChE (0.4 µM) in 100 mM sodium phosphate buffer (pH 7) at time zero. Rates were monitored by recording the increasing in absorption at 412 nm using a UV-VIS spectrometer.

Determination of Michaelis-Menten kinetics of AChE-pCBMA conjugates Acetylthiocholine iodide (0 – 100 µL of 10 mM in 100 mM sodium phosphate buffer (pH 7.4)) and 10 µL of DTNB solution (50 mM in DMSO) was mixed with 100 mM sodium phosphate buffer (980 - 880 µL, pH 7.4). Conjugate solution (10 µL, 5.5 µM of AChE) was added to the substrate solution. The initial substrate hydrolysis rate was monitored by recording the increase in absorbance at 412 nm using an UV-VIS absorbance spectrometer with a temperature-controlled cell holder at 37 °C. Michaelis-Menten parameters were determined by nonlinear curve fitting of initial rate versus substrate concentration plots using Enzfitter software.

Activity assays of Lyz-pCBMA conjugates Activity of Lyz-pCBMA conjugates was determined by two different substrates. Lyophilized *Micrococcus lysodeikticus* (Sigma Aldrich) was used to monitor enzymatic catalysis of cell wall lysis.[1] Absorption at 450 nm of suspended *M. lysodeikticus* (990 µL, 0.2 mg/mL) in 50 mM phosphate buffer (pH 6.0) was measured by UV-VIS spectrometer. 10 µL of Lyz-pCBMA solution (2.8 µM in 50 mM phosphate buffer (pH 6.0)) was added and the change of absorbance at 450 nm at room temperature was monitored.

p-nitrophenyl *β*-glycoside of *N*-acetylchitooliosaccharide [2] was also used to determine the activity of the conjugates. To the solution of 4-Nitrophenyl *β*-D-*N,N',N''*-triacetylchitotriose (10 µL of 50 mM in DMSO, Sigma Aldrich) in 50 mM phosphate buffer (980 µL, pH 6.0), conjugate solution (10 µL of 714 µM in 50 mM phosphate buffer, pH 6.0) was added and the absorption was measured at 405 nm using an UV-VIS absorbance spectrometer with a temperature-

controlled cell holder at 37 °C. The *p*-nitrophenol releasing rate was reported as hydrolysis activity of the conjugates.

Binding affinity of HABA to Avi-pCBMA conjugates 4'-hydroxyazobenzene-2-carboxylic acid (HABA, Sigma Aldrich) is a reagent that binds to Avidin and shows spectral changes, thus it is utilized to for determination of Avidin binding affinity.[3] Absorption at 500 nm of 300 µM HABA solution in phosphate buffered saline without calcium or magnesium (986 µL, Lonza) was measured using UV-VIS spectrometer. 16 µL of the conjugates solution (1.25 µM of Avidin in deionized water) was added to the HABA solution and incubated at room temperature for 1 min, and then absorption at 500 nm was measured. Change in absorbance at 500 nm was used to determine bound HABA to the conjugate.

Activity assay of Uox-pCBMA Enzymatic activity of the Uox-pCBMA conjugates was determined by oxidation of uric acid to allantoin.[4] Absorption at 290 nm of 50 µM uric acid in 20 mM sodium borate buffer (pH 8.5, 990 µL) was measured using an UV-VIS absorbance spectrometer with a temperature-controlled cell holder at 37 °C. Conjugate solution (10 µL, 57 µM of Uox in 20 mM borate buffer (pH 8.5)) was added to the substrate solution. The initial reaction velocity was monitored by recording the decrease in absorbance at 290 nm using the UV-VIS absorbance spectrometer at 37 °C. Activity of the conjugates (U/g) was determined from the initial velocity and concentration of Uox.

Impact of Agarase incubation for CT-pCBMA releasing from DMA beads To a suspension of obtained CT-pCBMA beads (100 µL) in 100 mM sodium phosphate (pH 6, 99 µL), agarose solution (1 µL, 1 U/µL) was added and rotated at room temperature for a given time (0 – 24 h). 20 mM sodium citrate (400 µL, pH 3) was added and rotated at room temperature for 1 h. The supernatant containing CT-pCBMA conjugate was separated from the beads by centrifugation.

Released active CT concentration in the supernatant was determined by an enzymatic activity assay on the hydrolysis of suc-AAPF-pNA using a standard curve with native CT.

A releasing study of CT-pCBMA was carried out by pre-incubation with Agarase to digest agarose[5] for quantitative recovery of the conjugate from the DMA beads. CT-pCBMA that was previously prepared on the DMA beads, was pre-incubated with Agarase (1 U/ 100 µL beads) in 100 mM sodium phosphate (pH 6) at room temperature for a designated time followed by incubation with 20 mM sodium citrate (pH 3). Released conjugates were quantified by an activity assay of hydrolysis of suc-AAPF-pNA. Agarase pre-incubation increased recovering the conjugate with incubation time (Supplementary Figure 23). We adopted the Agarase pre-incubation (1 U/100 µL of beads) in 100 mM sodium phosphate (pH 6) at room temperature overnight before adding 20 mM sodium citrate (pH 3) for releasing the conjugate from DMA beads.

Supplementary Discussion

Model Dye Binding and Release Studies

At pH 7 and 8, both the *N*-terminal and lysine mimic dyes quickly bound to the DMA beads, with maximum binding occurring within approximately 20 minutes. At pH 5 and 6, however, the binding rate of the *N*-terminal mimic, GGCy3, was an order of magnitude higher than that for the lysine mimic. Both GGCy3 and Cy5.5 amine showed increased initial binding rates with increased pH. At each pH 5 to 8 investigated, the initial binding rate of GGCy3 was higher than Cy5.5 amine indicating that the *N*-terminus α-amino had a higher binding affinity to the DMA beads, becoming even more pronounced at pH 5-6. Binding studies were performed by incubating the dye with the DMA beads at pH 5-8 over 60 minutes, washing with pH 8

phosphate buffer, releasing the dye at pH 3, and measuring fluorescence in the supernatant at various time points

Release studies were also performed using the GGCy3 and Cy5.5 amine fluorescent model dyes as a function of pH and time. Model dyes that were previously immobilized on the DMA beads at pH 8 were incubated in pH 3 to 6 releasing citrate buffers for 60 minutes. Supernatant fluorescence intensities were measured at various time points. Cy5.5 amine was rapidly released from the DMA beads with the maximum dye released within approximately 5 minutes at pH 3, 4, and 6 and within approximately 20 minutes at pH 5. GGCy3 also showed fast release at pH 3 and 4 within approximately 20 minutes, but slower release at pH 5 and 6. Interestingly, Cy5.5 amine showed an order of magnitude higher initial release rate than GGCy3 at all pH from 3 to 6. Also, the release rate for both model dyes decreased as pH increased

Trypsin Digestion

As observed with CT, tryptic digestion studies of the acetylcholinesterase-initiator complex showed that the peptide fragment containing the *N*-terminus (904.97 m/z, $[M+H]^+$) was absent when AChE was immobilized at pH 8.0 Analogous results were also obtained for the uricase-initiator complex. The peptide fragment containing the *N*-terminus was present in both native uricase and the uricase-initiator complex from the pH 6.0 experiment (676.97 m/z, $[M+H]^+$), but absent when the uricase-initiator was prepared after immobilization at pH 8.0. Avidin and lysozyme have *N*-terminii that are inaccessible, so we expected that they would not have site-specific reactions. Indeed, native avidin and avidin-initiator complex that had been reacted with DMA-agarose at both pH 6.0 or pH 8.0 retained the peptide fragment containing the *N*-terminus (555.99 m/z, $[2M+ACN+Na]^+$). This indicated that the *N*-terminus was not modified with the NHS-Br initiator under any of the reaction conditions. For lysozyme, since the *N*-terminus was a

lysine residue, we conducted 2-nitro-5-thiocyanatobenzoic (NTCB) acid digestion studies where NTCB reacted specifically with cysteine thiols by cyanylation. Similarly to avidin, the *N*-terminus peptide fragment (585.20 m/z, $[M+ACN+Na]^+$) in lysozyme was still free after immobilization at pH 6.0 and 8.0.

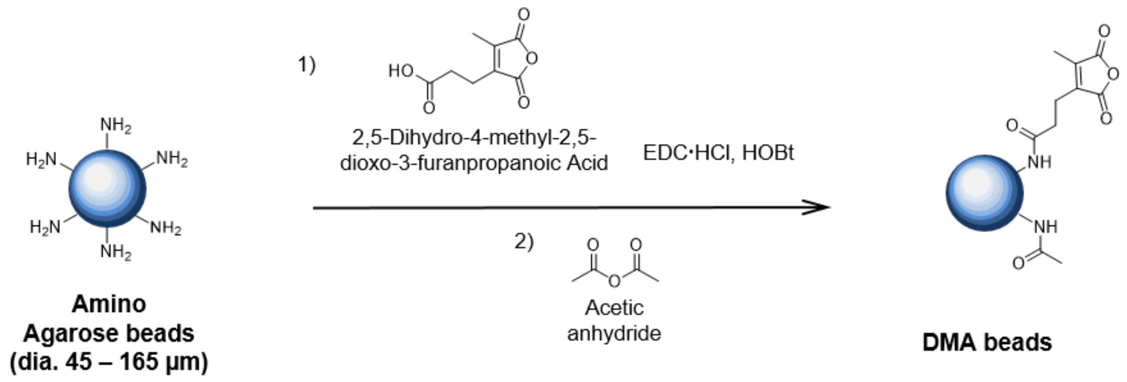

Supplementary Figure 1. Synthesis scheme of dialkyl maleic anhydride modified agarose beads.

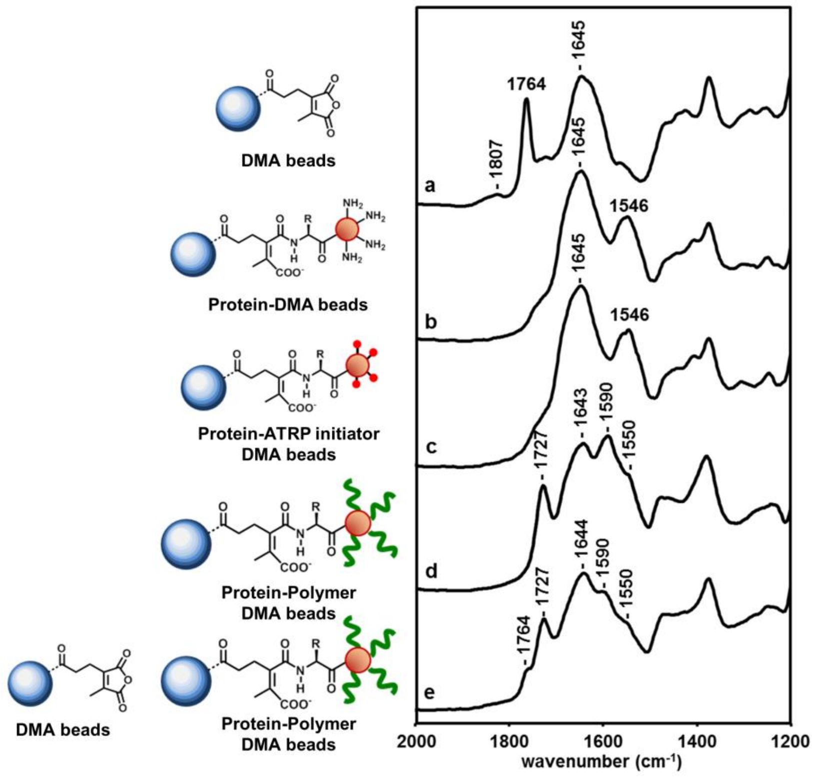

Supplementary Figure 2. FT-IR spectra of dried beads at each step of the PARIS synthesis a, DMA bead (1807 and 1764 cm^{-1} ($\nu_{C=O}$: anhydride) and 1647 cm^{-1} ($\nu_{C=N}$: imine and $\nu_{C=O}$: ester). **b,** CT immobilized DMA bead (1645 cm^{-1} ($\nu_{C=O}$: amide I), 1546 cm^{-1} ($\delta_{N\text{-}H}$: amide II)). **c,** CT-ATRP initiator on DMA bead. (1645 cm^{-1} ($\nu_{C=O}$: amide I), 1546 cm^{-1} ($\delta_{N\text{-}H}$: amide II)). **d,** CT-pCBMA conjugate on DMA bead 1727 cm^{-1} ($\nu_{C=O}$: ester), 1643 cm^{-1} ($\nu_{C=O}$: amide I), 1590 (ν_{as} :-COO^{-}), 1550 cm^{-1} ($\delta_{N\text{-}H}$: amide II)). **e,** DMA bead after CT-pCBMA cleavage (1764 cm^{-1}($\nu_{C=O}$: anhydride), 1727 cm^{-1} ($\nu_{C=O}$: ester), 1643 cm^{-1} ($\nu_{C=O}$: amide I), 1590 (ν_{as} :-COO^{-}), 1550 cm^{-1} ($\delta_{N\text{-}H}$: amide II)).

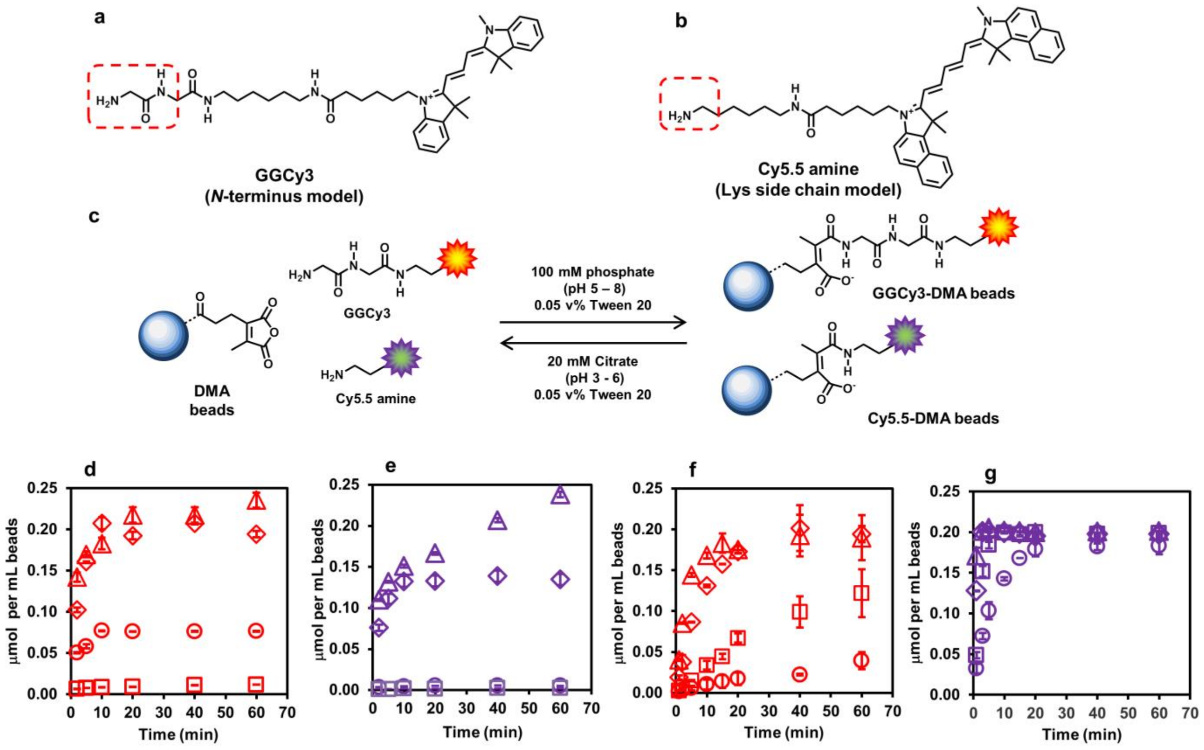

Supplementary Figure 3. pH dependence of binding and releasing affinities of GGCy3 and Cy5.5 amine to DMA-agarose beads a, Structure of Glycyl-glycyl-Cy3 as model for *N*-terminus. **b,** Structure of Cy5.5 amine as model for Lys side chain. **c,** Scheme for binding and releasing reaction between amino dyes and DMA beads. **d,** pH dependence of the binding rate of GGCy3 to DMA beads (pH 5, open square; pH 6, open circle; pH 7, open diamond; pH 8, open triangle). **e,** pH dependence of the binding rate of Cy5.5 amine to DMA beads (pH 5, open square; pH 6, open circle; pH 7, open diamond; pH 8, open triangle). **f,** pH dependence of the releasing rate of GGCy3 from DMA beads (pH 3, open triangle; pH 4, open diamond; pH 5, open square; pH 6, open circle). **g,** pH dependence of the releasing rate of Cy5.5 amine from DMA beads (pH 3, open triangle; pH 4, open diamond; pH 5, open square; pH 6, open circle). Error bars represent standard deviation from triplicate measurements.

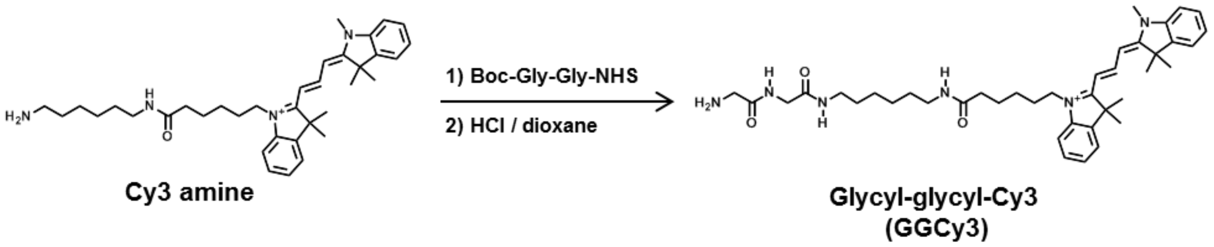

Supplementary Figure 4. Synthesis of N-terminus model dye glycyl-glycyl-Cy3.

Cy3 amine, HRMS (m/z): $[M\text{-}H]^+$ calcd. for $C_{36}H_{52}N_4O_5{}^{2+}$, 556.41; found, 556.84.

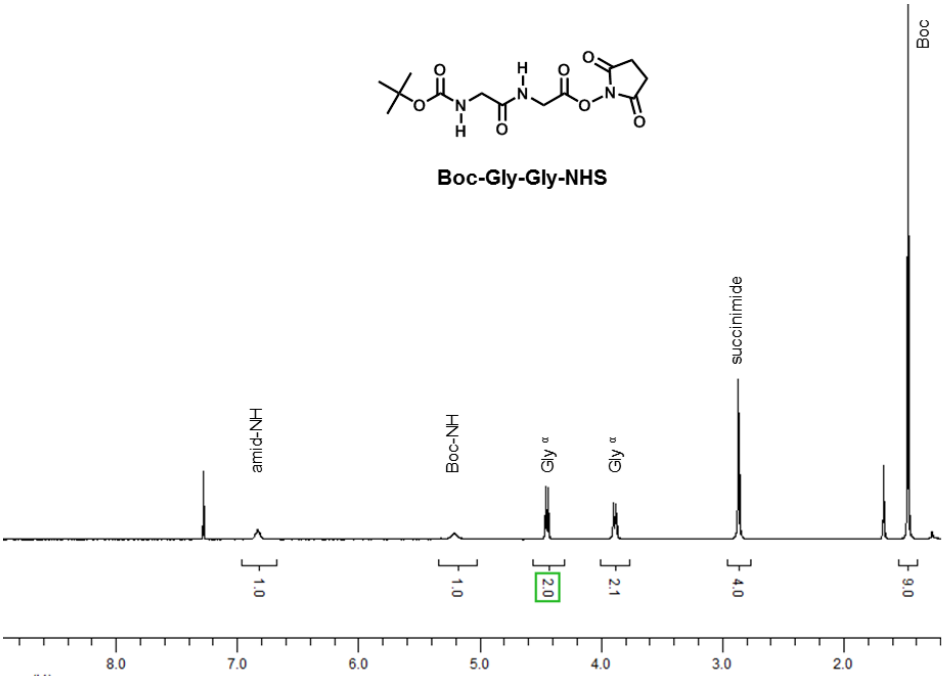

Supplementary Figure 5. ^{1}H NMR (300 MHz, CDCl3) spectrum of Boc-Gly-Gly-NHS.

Boc-Gly-Gly-NHS, mp: 139-142 °C**,** ^{1}H NMR (300 MHz, DMSO-d_6)δ 1.47 (s, 9 H, Boc-), 2.87 (s, 4 H, Succinimide), 3.88 (d, 2 H, J = 6.0 Hz, Gly$^{\alpha}$), 4.45 (d, 2 H, , J = 6.0 Hz, Gly$^{\alpha'}$), 5.21 (broad s, 1 H, NH) and 6.83 (broad s, 1 H , NH) ppm; ^{13}C NMR (75 MHz, DMSO-d_6) δ 24.8, 25.0, 27.0, 27.6, 27.8, 28.4, 37.7, 42.5, 77.7, 155.3, 165.8, 169.5, 169.8, 172.3 ppm; IR (KBr pellet) 2980, 2938, 1821, 1789, 1741, 1702, 1665, 1575 and 1514 cm^{-1}.

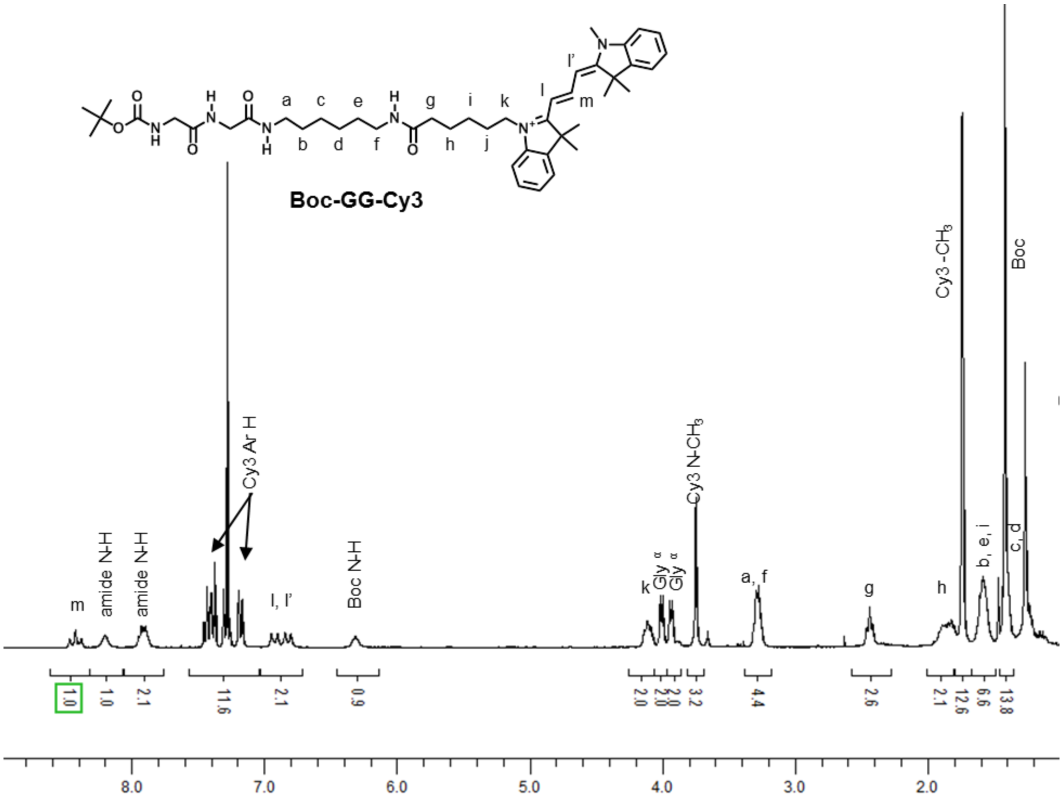

Supplementary Figure 6. ^{1}H NMR (300 MHz, CDCl$_3$) spectrum of Boc-GG-Cy3.

Boc-Gly-Gly-Cy3, oily compound**,** ^{1}H NMR (300 MHz, CDCl$_3$) δ 1.41 (m, 9 H, Boc- and 4 H, 2 × CH$_2$), 1.58 (m, 6 H, 3 × CH$_2$), 1.73 (s, 12 H, Cy3-CH$_3$), 1.83 (m, 2 H, CH$_2$), 2.42 (t, J = 7.2 Hz, C=OCH$_2$), 3.27 (m, 4 H, 2 × NHCH2), 3.74 (s, 3 H, Cy3 N-CH$_3$), 3.92 (d, 2 H, J = 5.7 Hz, Gly$^{\alpha}$), 3.99 (d, 2 H, , J = 5.7 Hz, Gly$^{\alpha'}$), 6.25 (broad s, 1 H, Boc-NH), 6.82 (d, 1 H, J = 13.2 Hz, C=CH), 6.92 (d, 1 H, J = 13.2 Hz, C=CH), 7.16 – 7.45 (m, 8 H, Cy3-Ar H), 7.90 (m, 2 H, amide), 8.15 (broad t, 1 H, J = 4.2 Hz, amide), 8.41 (t, 1 H, J = 13.2 Hz, CH=CH-CH) ppm; ^{13}C NMR (75 MHz, DMSO-d$_6$) δ 25.0, 25.5, 26.0, 26.7, 26.8, 27.7, 27.8, 28.2, 28.5, 28.8, 35.6, 35.7, 38.5, 42.9, 44.3, 48.8, 48.9, 79.3, 103.2, 111.1, 121.5, 122,5 125.2, 125.6, 128.0, 128.9, 140.0, 141.5, 142.2, 150.2, 156.0, 169.2, 170.4, 173.4, 173.8 ppm; IR (NaCl plate) 2950, 2925, 2856, 1655, 1559, 1493, 1458, 1415 and 1371 cm^{-1}; HRMS (m/z): [M-H]$^{+}$ calcd. for $C_{45}H_{65}N_6O_5$, 769.501; found, 770.051.

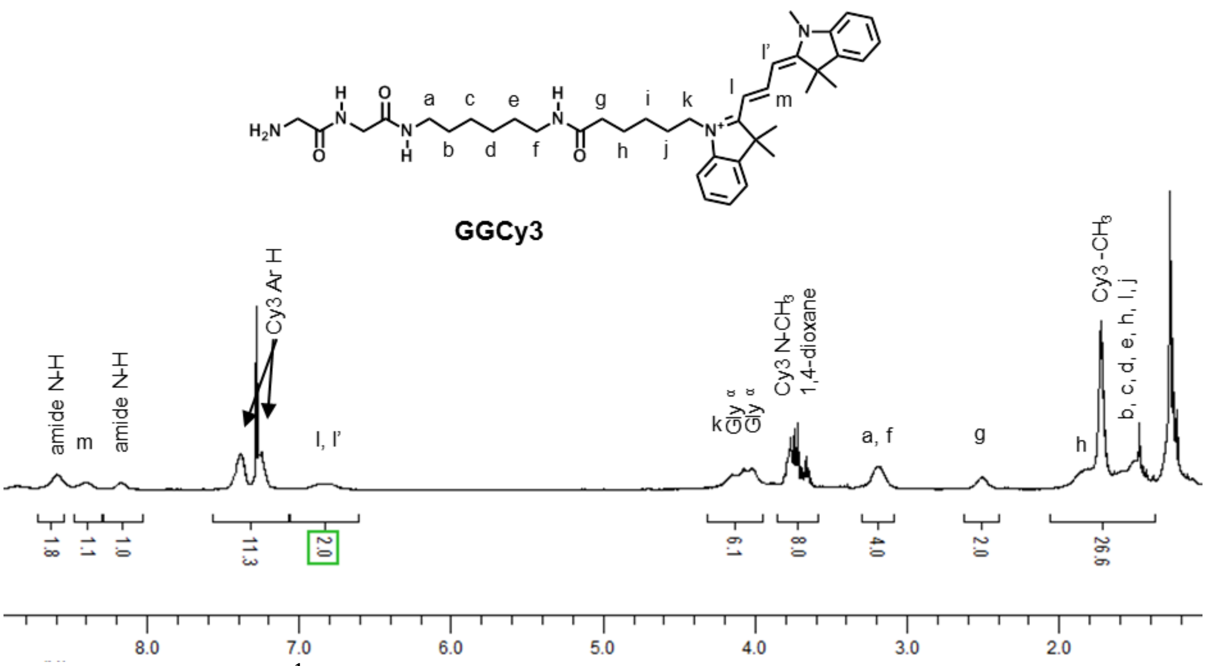

Supplementary Figure 7. ^{1}**H NMR (300 Mhz, CDCl**$_3$**) spectrum of GGCy3.**

Gly-Gly-Cy3, oily compound, ^{1}H NMR (300 MHz, $CDCl_3$) δ 1.4 – 1.9 (broad, 14 H, 7 × CH_2 and 12 H, Cy3-CH_3), 2.5 (broad, 2 H, CH_2), 3.2 (broad, 4 H, 2 × CH_2), 3.8 (broad, 3 H, Cy3 N-CH_3), 4.0 – 4.3 (broad, 2 H, CH_2 and 4 H, 2 × Gly$^{\alpha}$), 6.8 (broad, 2 H, CH=CH), 7.3 and 7.4 (broad, 8 H Cy3-Ar H), 8.2 (broad, 1 H, amide), 8.4 (broad, 1 H, CH=CH-CH), 8.6 (broad, 2 H, amide) ppm; ^{13}C NMR (75 MHz, DMSO-d_6) δ 25.2, 25.7, 26.1, 26.8, 28.0, 28.2, 28.7, 28.9, 29.0, 29.1, 29.7, 34.0, 35.0, 36.1, 38.7, 39.7, 42.9, 49.0, 49.2, 103.9, 105.6, 116.0, 125.7, 127.5, 128.5, 131.2, 140.5, 141.9, 169.3, 170.1, 173.8 ppm; IR (NaCl plate) 2956, 2924, 2853, 1712, 1651, 1557, 1493, 1456, 1416 and 1376 cm^{-1}; HRMS (m/z): $[M-2H]^+$ calcd. for $C_{40}H_{57}N_6O_3^{2+}$, 670.46; found, 670.94.

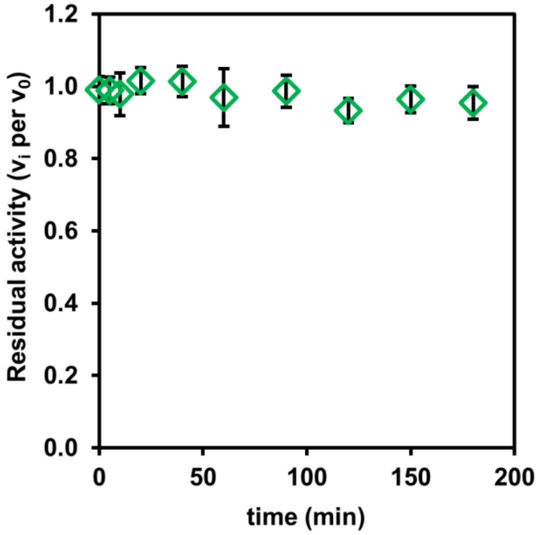

Supplementary Figure 8. Native chymotrypsin (CT) stability in 20 mM citrate buffer (pH 3) at 25 °C. Chymotrypsin's residual activity was measured at pH 8 (100 mM sodium phosphate buffer) using suc-AAPF-pNA as a substrate at specified time points after incubation in pH 3 releasing buffer. Chymotrypsin maintained full activity even after 3 hours indicating that the lower pH of releasing buffer did not cause enzymatic activity loss. Error bars represent standard deviation from triplicate measurements.

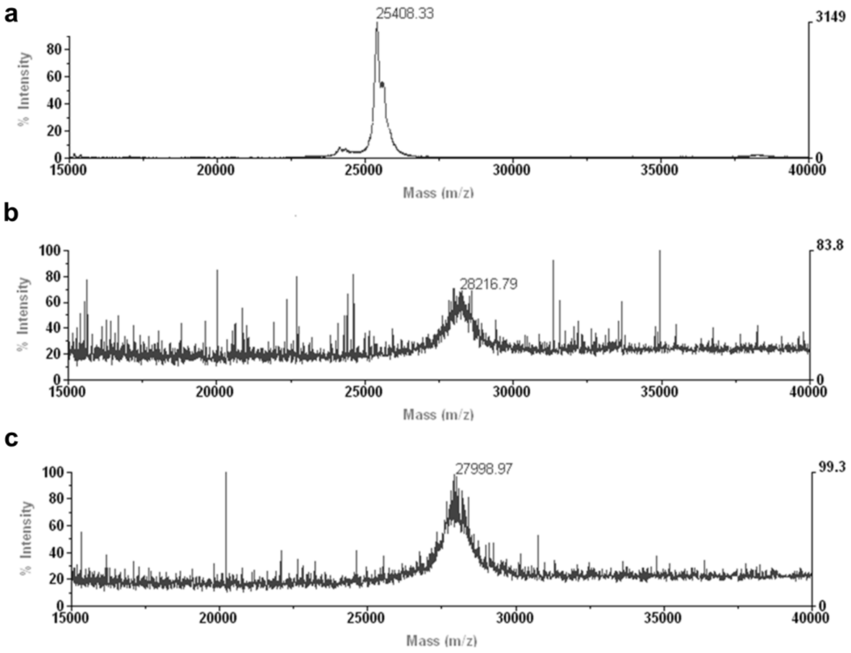

Supplementary Figure 9. MALDI-ToF mass spectroscopy of native CT and initiator modified CT (CT-Br) initially immobilized at pH 6.0 and pH 8.0 to determine number of modifications. A, Spectrum of native CT. CTBr was modified with **b,** 13 initiators at pH 6.0 and **c,** 11 initiators at pH 8.0.

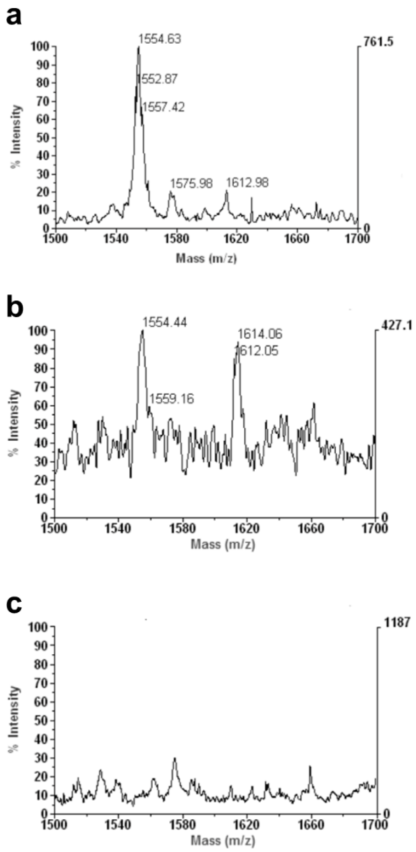

Supplementary Figure 10. Determination of N-terminus selectivity. The presence of *N*-terminus peptide fragment at 1554.63 m/z in the spectra for **a,** native CT and **b,** CT-initiator immobilized at pH 6.0 combined with the disappearance of the same peptide fragment in the spectrum for **c,** CT-initiator immobilized at pH 8.0 indicated *N*-terminal selective binding to the DMA beads.

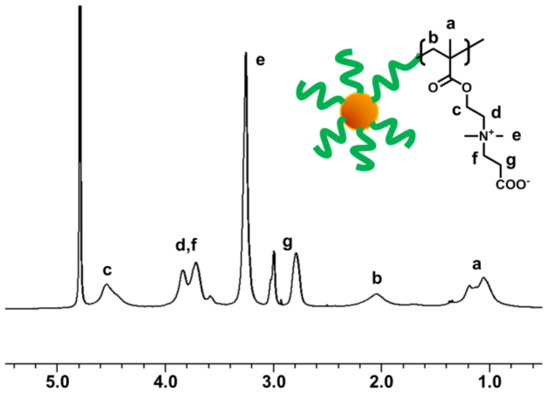

Supplementary Figure 11. **^{1}H NMR spectrum of released CT-pCBMA by PARIS in *D_2*O to verify polymer chemical structure.**

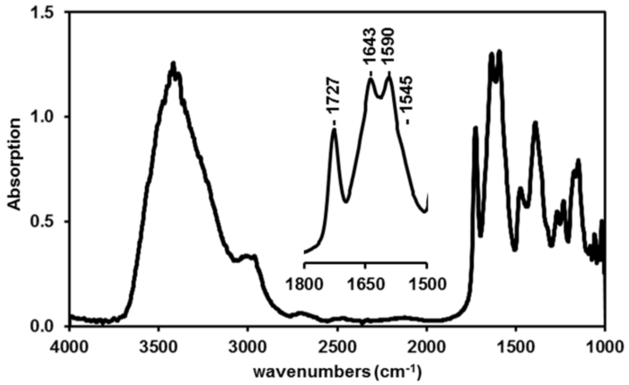

Supplementary Figure 12. FT-IR spectrum of released CT-pCBMA by PARIS. Spectrum was measured by the method using KBr pellet.

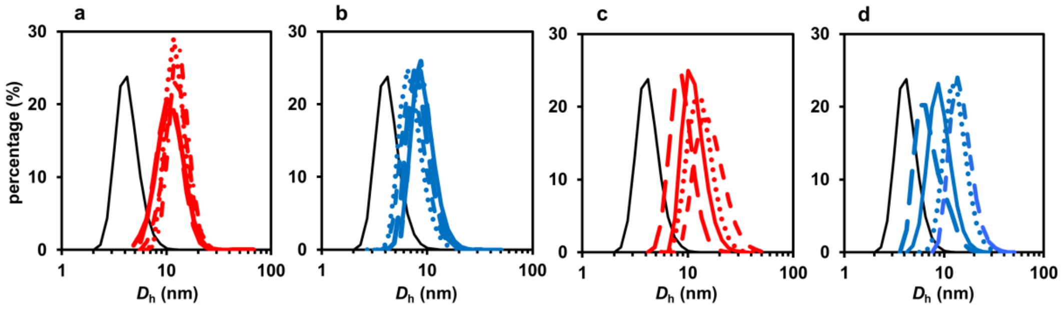

Supplementary Figure 13. Particle size distribution of CT-pCBMA conjugates by number. **a,** fixed monomer concentration of 25 mM by solution method (polymerization time for 5 min, red dot; 10 min, red dash dot; 20 min, red dash; 40 min, red long dash; 60 min, red solid; native CT, black solid). **b,** fixed monomer concentration of 25 mM by PARIS (5 min, blue dot; 10 min, blue dash dot; 20 min, blue dash; 40 min, blue long dash; 60 min, blue solid; native CT, black solid). **c,** increasing monomer concentration for a 60 min reaction time by solution method (12.5 mM, red dash; 25 mM, red solid; 50 mM, red dot; 100 mM, red dash). **d,** increasing monomer concentration for a 60 min reaction time by PARIS (12.5 mM, blue dash; 25 mM, blue solid; 50 mM, blue dot; 100 mM, blue dash).

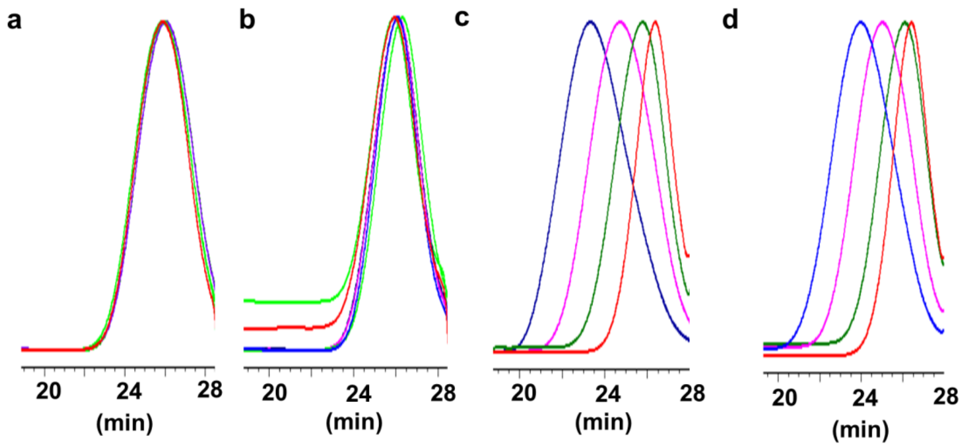

Supplementary Figure 14. GPC traces of cleaved pCBMA from CT-pCBMA conjugates. **a,** fixed monomer concentration of 25 mM by solution method. **b,** fixed monomer concentration of 25 mM by PARIS. (for **a** and **b** reaction time: green, 5 min; red, 10 min; black, 20 min; purple, 40 min; blue, 60 min) **c,** increasing monomer concentration for a 60 min reaction time by solution method. **d,** increasing monomer concentration for a 60 min reaction time by PARIS (for **c** and **d** monomer concentration: red, 12 mM; green, 25 mM; purple, 50 mM; blue, 100 mM).

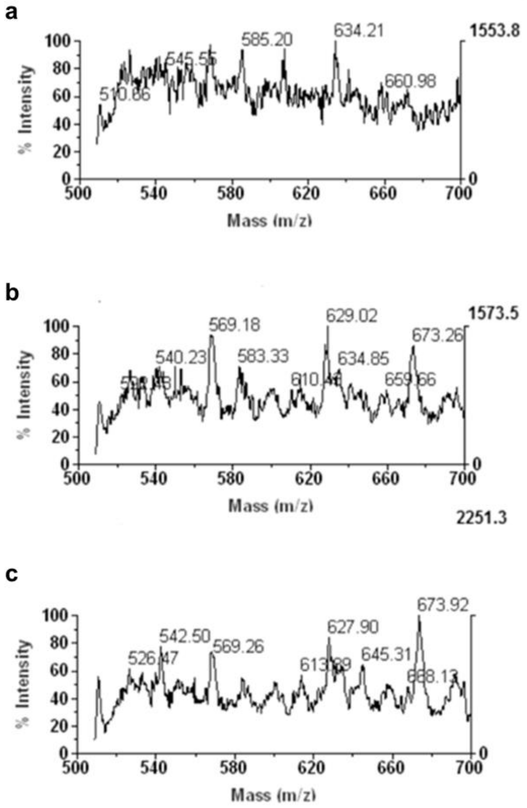

Supplementary Figure 15. The presence of *N*-terminus peptide fragment at 585.20 m/z in the spectra for a, native Lysozyme and **b,** Lysozyme-initiator immobilized at pH 6.0 and **c,** Lysozyme-initiator immobilized at pH 8.0 suggested that the *N*-terminus did not react with either the DMA beads or the bromine-initiator. Exposed surface accessibility calculations showed that the α-amino group was buried and was most likely unable to react.

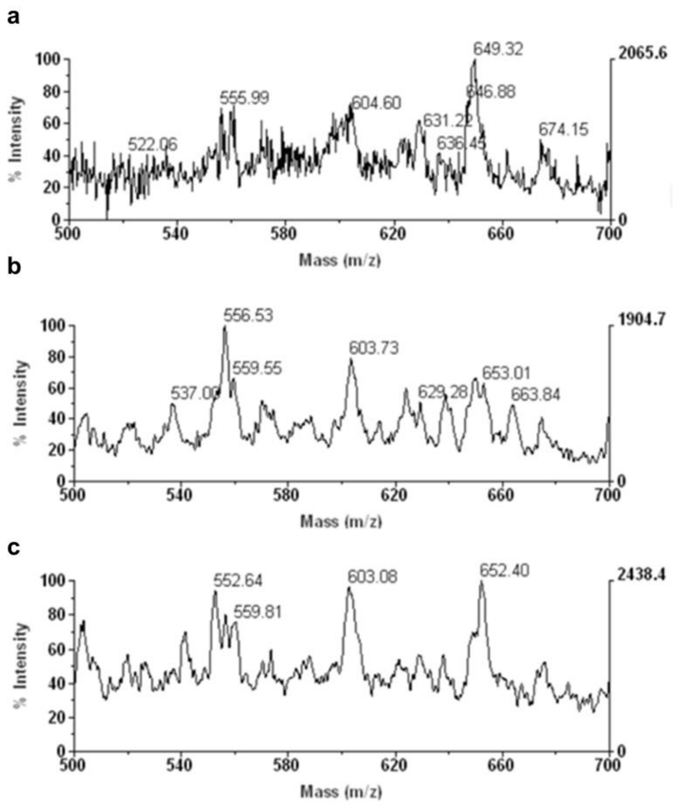

Supplementary Figure 16. The presence of *N*-terminus peptide fragment at 555.99 m/z in the spectra for a, native Avidin and **b,** Avidin-initiator immobilized at pH 6.0 and **c,** Avidin-initiator immobilized at pH 8.0 suggested that the *N*-terminus did not react with either the DMA beads or the bromine-initiator. Exposed surface accessibility calculations showed that the α-amino group was buried and was most likely unable to react.

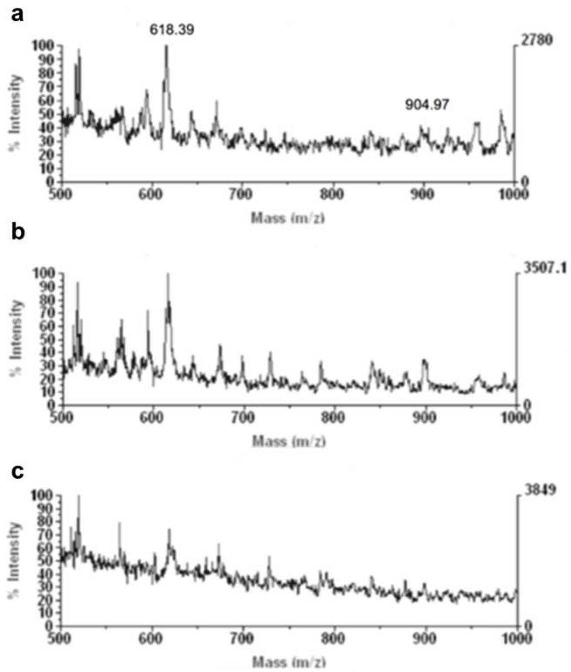

Supplementary Figure 17. The presence of *N*-terminus peptide fragment at 904.97 m/z in the spectra for a, native AChE and **b,** AChE-initiator immobilized at pH 6.0 combined with the disappearance of the same peptide fragment in the spectrum for **c,** AChE-initiator immobilized at pH 8.0 indicated *N*-terminal selective binding to the DMA beads.

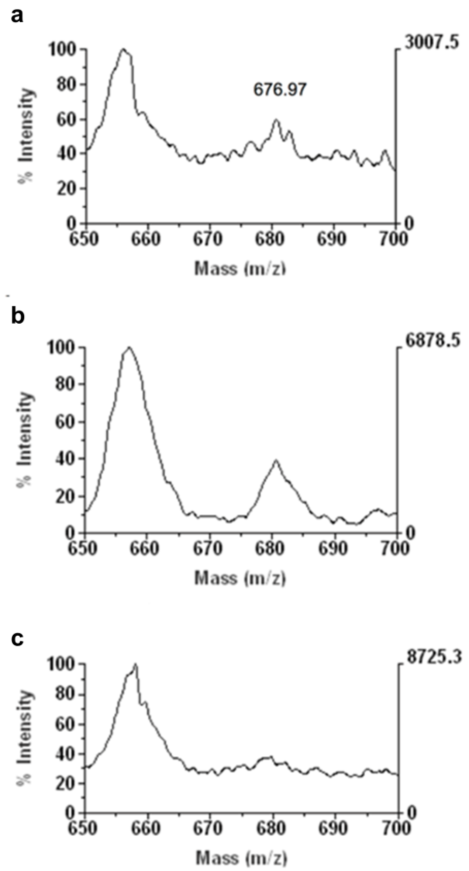

Supplementary Figure 18. The presence of *N*-terminus peptide fragment at 676.97 m/z in the spectra for a, native Uox and **b,** Uox-initiator immobilized at pH 6.0 combined with the disappearance of the same peptide fragment in the spectrum for **c,** Uox-initiator immobilized at pH 8.0 indicated *N*-terminal selective binding.

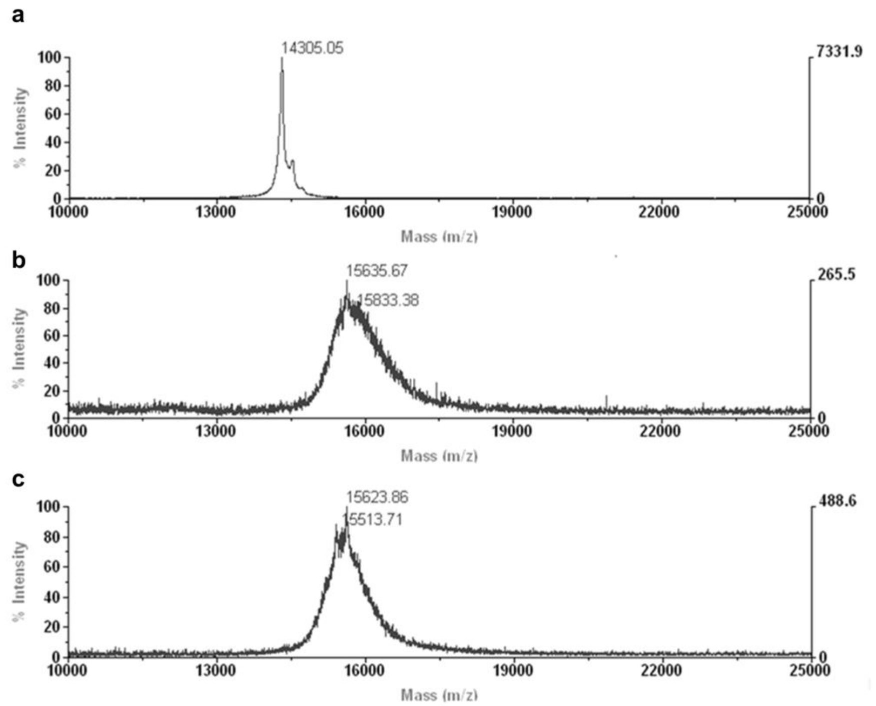

Supplementary Figure 19. MALDI-ToF mass spectroscopy of native lysozyme and initiator modified lysozyme (Lyz-Br) initially immobilized at pH 6.0 and pH 8.0 to determine number of modifications. a, native lysozyme **b,** lysozyme-initiator immobilized at pH 6.0 (6 initiators) and **c,** lysozyme-initiator immobilized at pH 8.0 (6 initiators).

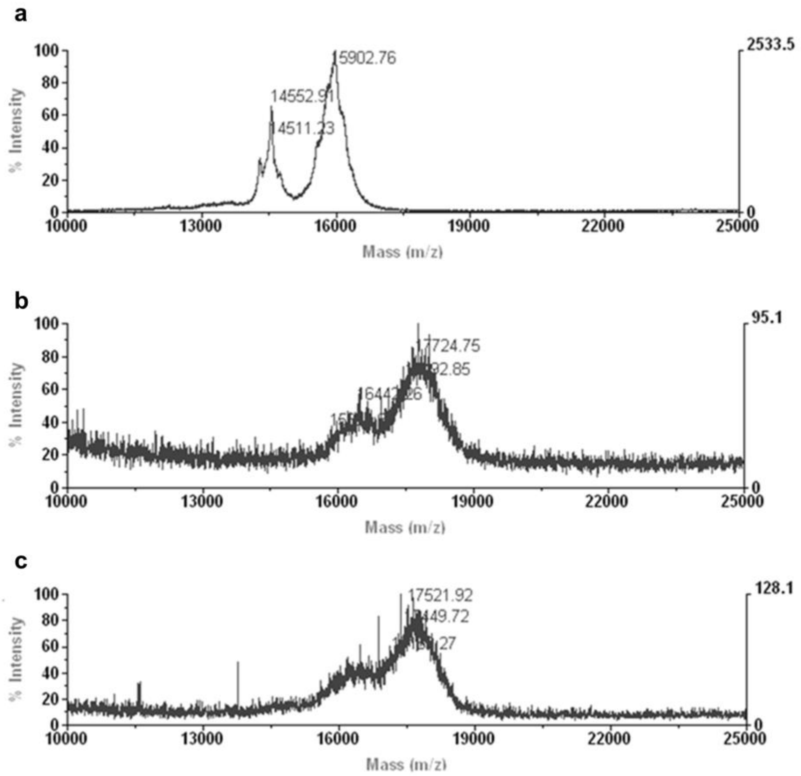

Supplementary Figure 20. MALDI-ToF mass spectroscopy of native avidin and initiator modified avidin (Av-Br) initially immobilized at pH 6.0 and pH 8.0 to determine number of modifications. a, native avidin **b,** avidin-initiator immobilized at pH 6.0 (8 initiators) and **c,** avidin-initiator immobilized at pH 8.0 (8 initiators).

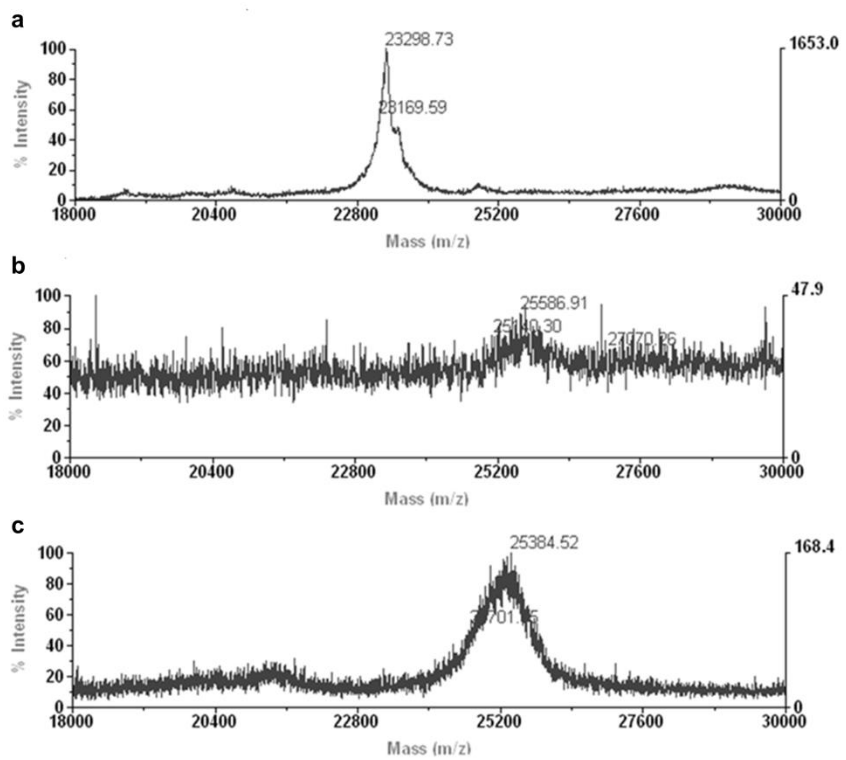

Supplementary Figure 21. MALDI-ToF mass spectroscopy of native acetylcholinesterase and initiator modified acetylcholinesterase (AChE-Br) initially immobilized at pH 6.0 and pH 8.0 to determine number of modifications. a, native AChE $[M+3H]^{3+}$ **b,** AChE-initiator immobilized at pH 6.0 (11 initiators) and **c,** AChE-initiator immobilized at pH 8.0 (9 initiators).

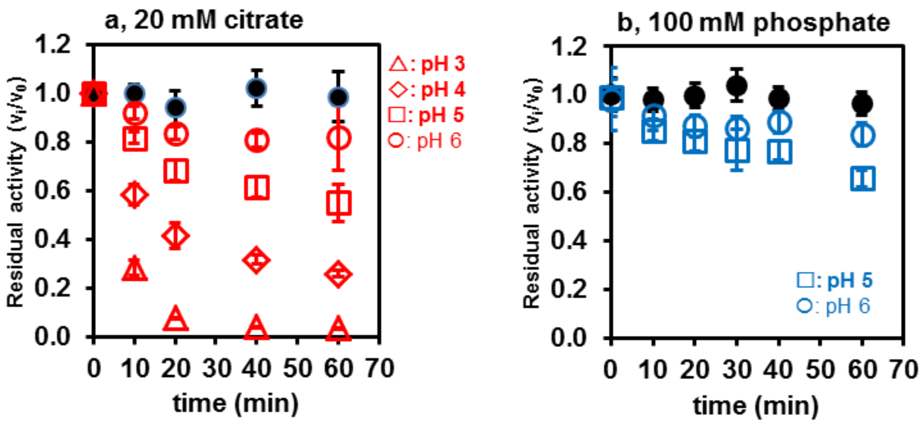

Supplementary Figure 22. Irreversible inactivation of AChE in low pH a, incubation in 20 mM citrate buffer (pH 3 – 6) at room temperature. **b,** incubation in 100 mM sodium phosphate buffer (pH 5 and 6) at room temperature. Error bars represent standard deviation from triplicate measurements.

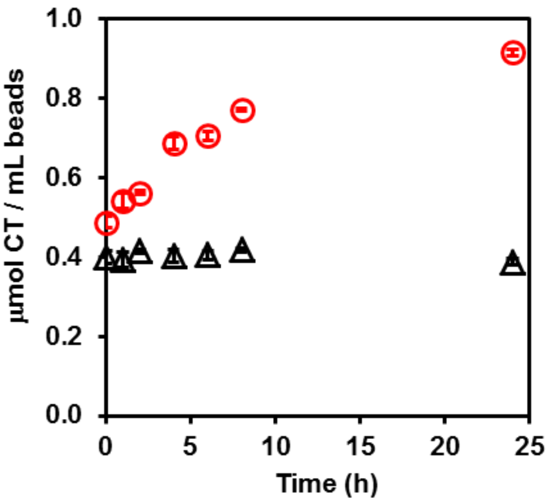

Supplementary Figure 23. Impact of Agarase pre-incubation on CT-PCBA releasing from the DMA beads. Pre-incubation with Agarase , red open circle; without Agarase pre-incubation, black open triangle. Error bars represent standard deviation from triplicate measurements.

Supplementary Table 1. pH dependence of initial binding and release rate of GGCy3 and Cy5.5 amine to DMA beads.

Initial Binding Rate (μmol $min^{-1}·mL^{-1}$ beads)			Initial Release Rate (μmol $min^{-1}·mL^{-1}$ beads)		
**pH**	**GGCy3**	**Cy5.5 amine**	**pH**	**GGCy3**	**Cy5.5 amine**
5	6.5 ± 0.1	0.4 ± 0.1	3	44.4 ± 1.2	170.7 ± 10.1
6	25.3 ± 0.4	1.7 ± 0.2	4	18.8 ± 2.3	127.8 ± 5.3
7	51.3 ± 1.4	37.9 ± 1.9	5	2.9 ± 0.2	48.5 ± 3.7
8	71.1 ± 0.2	54.8 ± 4.2	6	1.2 ± 0.1	32.1 ± 5.7

Supplementary Table 2. Peak analysis of peptide fragments after trypsin digestion or NTCB digestion.

Protein	Peptide Fragment	Expected mass (m/z)	Observed mass (m/z)
Chymotrypsin	[1]CGVPAIQPVLSGLSR	1554.78 $[M+H]^+$	1554.63
Acetylcholinesterase	[1]SELLVNTK	904.05 $[M+H]^+$	904.97
Uricase	[1]MAHYR	677.79 $[M+H]^+$	676.97
Avidin	[1]AR	554.58 $[2M+ACN+Na]^+$	555.99
Lysozyme	[1]KVFGR	586.60 $[M+ACN+Na]^+$	585.20

Supplementary Table 3. Michaelis-Menten kinetic values for AChE-catalyzed hydrolysis of acetylthiocholine iodide.

AChE-pCBMA	k_{cat} (s^{-1})	K_M (mM)	k_{cat}/K_M (mM^{-1} s^{-1})	Ratio of enzymatic efficiency (PARIS:solution)
PARIS	0.89 ± 0.02	0.179 ± 0.010	4.97 ± 0.30	1.09 ± 0.08
Solution	1.30 ± 0.02	0.285 ± 0.011	4.56 ± 0.19	

Supplementary Table 4. Enzymatic activity of Lyz-pCBMA.

	M. lysodeikticus		*N*-acetyl *β*-chitotriose	
	ΔA_{450} min^{-1} (× 10^{-5})	Ratio of lysis (PARIS:solution)	ΔA_{405} min^{-1} (× 10^{-5})	Ratio of hydrolysis (PARIS:solution)
PARIS	91.9 ± 0.2	1.94 ± 0.13	3.60 ± 0.65	1.95 ± 0.53
solution	47.3 ± 1.9		1.85 ± 0.56	

Supplementary Table 5. Binding affinity of HABA to Avi-pCBMA conjugates.

Avi-pCBMA	ΔA_{500}	Bound HABA (μM)	Ratio of binding affinity of HABA (PARIS:solution)
PARIS	0.0316 ± 0.003	0.93 ± 0.13	1.01 ± 0.14
solution	0.0313 ± 0.001	0.92 ± 0.04	

$[Avi]_0$ = 2 μM, ε_{500} = 34000 M^{-1}

Supplementary Table 6. Enzymatic activity of Uox-pCBMA conjugates.

Uox-pCBMA	ΔA_{290} min^{-1} ($\times 10^{-5}$)	U g^{-1}	Ratio of activity (PARIS:solution)
PARIS	17.2 ± 1.10	0.70 ± 0.05	2.33 ± 0.15
solution	7.45 ± 2.75	0.30 ± 0.11	

ε_{290} = 12300 M^{-1}

Supplementary References

1. Smolelis, A. N. & Hartsell, S. E., The determination of lysozyme. *J. Bacteriol.* **58**, 731-736 (1949).
2. Nanjo, F., Sakai, K. & Usui, T., *p*-Nitrophenyl penta-*N*-acetyl-β-chitopentaoside as novel synthetic substrate for the colorimetric assay of lysozyme. *J. Biochem*. **104**, 255-258 (1998).
3. Green, N. M., A spectrophotometric assay for avidin and biotin based on binding of dyes by avidin. *Biochem. J.*, **94** 23-24, (1965).
4. Mahler, H. R. *et al.* Studies on uricase I. Preparation, purification, and properties of a cuproprotein. *J. Biol. Chem.*, **216**, 625-641 (1955).
5. Yaphe, W., The use of agarase from *Pseudomonas atlantica* in the identification of agar in marine algae (Rhodophyceae). *Can. J. Microbiol.* **3**, 987-993 (1957).
